# The effects of FES cycling combined with virtual reality racing biofeedback on voluntary function after incomplete SCI: a pilot study

**DOI:** 10.1186/s12984-019-0619-4

**Published:** 2019-11-27

**Authors:** Lynsey D. Duffell, Sue Paddison, Ahmad F. Alahmary, Nick Donaldson, Jane Burridge

**Affiliations:** 1 Department of Medical Physics & Biomedical Engineering, University College London, Malet Place Engineering Building, Gower Street, London, WC1E 6BT UK; 2 London Spinal Cord Injury Centre, Royal National Orthopaedic Hospital, Stanmore, UK; 3 Faculty of Environmental and Life Sciences, University of Southampton, Southampton, UK

**Keywords:** Biofeedback, Cycling, Functional electrical stimulation, ISNC-SCI motor score, Spinal cord injury, Virtual reality

## Abstract

**Background:**

Functional Electrical Stimulation (FES) cycling can benefit health and may lead to neuroplastic changes following incomplete spinal cord injury (SCI). Our theory is that greater neurological recovery occurs when electrical stimulation of peripheral nerves is combined with voluntary effort. In this pilot study, we investigated the effects of a one-month training programme using a novel device, the iCycle, in which voluntary effort is encouraged by virtual reality biofeedback during FES cycling.

**Methods:**

Eleven participants (C1-T12) with incomplete SCI (5 sub-acute; 6 chronic) were recruited and completed 12-sessions of iCycle training. Function was assessed before and after training using the bilateral International Standards for Neurological Classification of SCI (ISNC-SCI) motor score, Oxford power grading, Modified Ashworth Score, Spinal Cord Independence Measure, the Walking Index for Spinal Cord Injury and 10 m-walk test. Power output (PO) was measured during all training sessions.

**Results:**

Two of the 6 participants with chronic injuries, and 4 of the 5 participants with sub-acute injuries, showed improvements in ISNC-SCI motor score > 8 points. Median (IQR) improvements were 3.5 (6.8) points for participants with a chronic SCI, and 8.0 (6.0) points for those with sub-acute SCI. Improvements were unrelated to other measured variables (age, time since injury, baseline ISNC-SCI motor score, baseline voluntary PO, time spent training and stimulation amplitude; *p* > 0.05 for all variables). Five out of 11 participants showed moderate improvements in voluntary cycling PO, which did not correlate with changes in ISNC-SCI motor score. Improvement in PO during cycling was positively correlated with baseline voluntary PO (R^2^ = 0.50; *p* < 0.05), but was unrelated to all other variables (*p* > 0.05). The iCycle was not suitable for participants who were too weak to generate a detectable voluntary torque or whose effort resulted in a negative torque.

**Conclusions:**

Improved ISNC-SCI motor scores in chronic participants may be attributable to the iCycle training. In sub-acute participants, early spontaneous recovery and changes due to iCycle training could not be distinguished. The iCycle is an innovative progression from existing FES cycling systems, and positive results should be verified in an adequately powered controlled trial.

**Trial registration:**

ClinicalTrials.gov, NCT03834324. Registered 06 February 2019 - Retrospectively registered, https://clinicaltrials.gov/ct2/show/NCT03834324. Protocol V03, dated 06.08.2015.

## Background

Neurological recovery following Spinal Cord Injury (SCI) is thought to take place during the first few months following injury. Later, functional improvement may be due to normal motor learning and muscle strengthening [1]. Factors that are critical to neuroplasticity are timing and intensity of therapy [2, 3]. Non-primate studies have shown that neural re-organisation occurs after 400 repetitions of a reaching movement but not 60 [4], such intensity is unlikely to be achieved through conventional therapy. Rehabilitation technologies are becoming more widely used to increase number of repetitions, for example partial bodyweight support treadmill training and robotic devices. Despite their theoretical potential, evidence for effectiveness is equivocal [5] and cost-effectiveness is limited because they are expensive and require physiotherapists’ support.

Functional Electrical Stimulation (FES)-cycle ergometry is a low-cost, potentially home-usable alternative [6]. FES is a means of producing contractions in muscles and is applied via electrodes either on the skin or implanted. FES has been found to have a therapeutic or “carry-over” effect on gait re-education and lower limb strengthening in studies with stroke and SCI patients [7–10]. Ambrosini et al. [11] tested FES cycling exercise in a double-blind randomised trial as a therapy after stroke: FES cycling was compared with a placebo intervention (passive cycling with electrodes attached without delivering any stimulation current). The FES participants showed statistically significant improvements in motor power of the paretic lower extremity compared with placebo subjects. FES participants also experienced improved walking speed, which was maintained 4 months after the treatment, however this parameter was not statistically significantly different compared with the placebo group.

McDonald et al. [12] described a single case study of functional recovery after a 3-year program of activity (“activity-based recovery”) incorporating FES muscle conditioning and FES cycling: the individual improved from ASIA Imapirment Scale (AIS) Grade A to AIS Grade C. Later, the same group reported a study with 45 chronic subjects, 25 of whom used FES for between 3 and 168 months (mean 29.5 months) and 20 controls, matched by age, gender, injury level, injury severity, and duration of injury, who received range of motion and stretching. The average functional improvements in International Standards for Neurological Classification of SCI (ISNC-SCI) motor score were 8.1 (SD 10.0) for the FES group, which they defined as a clinically important gain in neurological function, and 0.6 (SD 6.5) for the controls [13].

Yaşar et al [14] used FES-cycling to train 10 people with incomplete (AIS C or D) SCI who were all able to walk 10 m with a cane or walker (all were between 24 and 33 months post-injury). Their outcome measures included ISNC-SCI motor score. The therapy sessions were of 1 h, 3 times per week for 3 months and outcome measures were made at the end of the therapy and again 3 months later. The participants were instructed *not* to make any voluntary effort. Average motor scores improved by 1.7 after the therapy and 4.7 at follow up. There were also significant improvements in spasticity, Functional Independence Measure (FIM) and oxygen consumption while walking. However, these authors commented that there was no control group so the improvement might not have been due to the electrical stimulation.

Greater changes in corticospinal transmission, have been demonstrated when nerve stimulation from two different sources is synchronised, (termed associated stimuli) for example repetitive Transcranial Magnetic Stimulation (TMS) of the motor cortex paired with peripheral stimulation of the common peroneal (to cause contraction of the ankle dorsiflexor muscles) rather than when each stimulation is provided asynchronously [15, 16]. This observation may explain why FES during walking in patients with central nervous system lesions, has a therapeutic effect when stimulation of the common peroneal nerve coincides with the person’s natural attempt to dorsiflex their ankle – i.e. voluntary drive from the motor cortex [17]. The mechanism is not fully understood, but could be due to neuroplastic changes within the central nervous system [18–20], involving a Hebbian type learning effect from concurrent voluntary neural drive and electrical muscle stimulation: antidromic electrical impulses may raise the resting potential of the anterior horn cells, so that they are more likely to discharge thus providing pre and post synaptic activity and ‘learning opportunities’ when accompanying voluntary activity [18]. Most FES cycles and motor-assisted ergometers (e.g. RT300) do not capitalise on this effect because no incentive is given to encourage the patient to use voluntary effort to turn the pedals.

To our knowledge, voluntary intention, combined with stimulation during purposeful cycling, and providing feedback based only on the voluntary effort, has not been tested. This may be because of the technical difficulty of reliably separating voluntary effort from FES-activated muscle. In earlier work in our lab, we attempted to estimate the torque from the quadriceps electromyography (EMG) in able-bodied subjects while stimulating the muscle and blanking the stimulus artefact, but because of the high variability of the RMS value, this averaging had to be continued for many revolutions of the pedals to reach a sufficient confidence level, and therefore could not be used for real-time biofeedback where the speed must quickly respond to changes in voluntary effort [21]. Therefore we tackled the problem differently. Stimulation was applied only on alternate revolutions of the pedals so that the average torque during the non-stimulation revolution represented voluntary effort*.* It was assumed that the effort was the same during revolutions with stimulation and those without. In order to maximise voluntary effort, the torque signal from non-stimulated revolutions was used as the input to a commercially available virtual reality (VR) cycle racing game. This approach avoids the practical complication of EMG, and the differences between patients in the muscles paralysed by iSCI. It is essential for practical home-use of the iCycle.

**Fig. 1:**
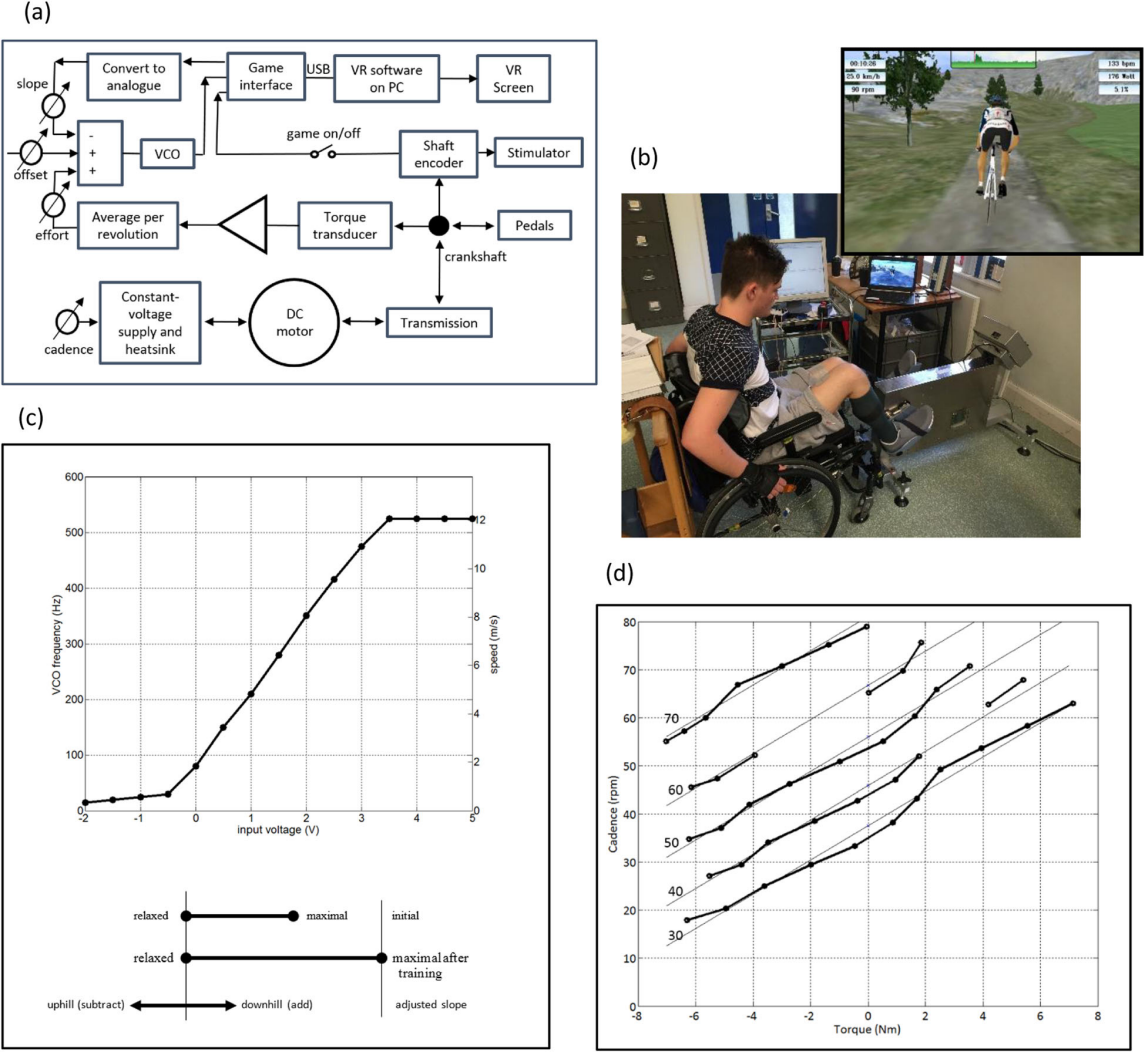
**a** Block Diagram of iCycle. The cadence control sets the voltage applied to the DC motor. The measured torque-cadence curves are shown in (**d**); the stiffness was made intentionally low to avoid musculo-skeletal damage. When the torque is positive, the power supply is absorbing power which is dissipated in a heat sink. Stimulation was applied during alternate revolutions of the pedals and the output of the torque transducer is averaged over revolutions without stimulation to give the signal called *effort*. For each of the six stimulation channels, the output was gated so that the 30 Hz pulses were applied from a *switch-on* to a *switch-off* angle, which could be set for each participant. A commercial virtual reality cycling game was adapted for the iCycle. The hardware interface for this game has an output which is approximately the slope of the road in the rolling scenery. This *slope* is subtracted from the *effort* and the difference frequency-modulated by a voltage-controlled oscillator (VCO) before being fed back as the *wheelspeed.* There is also a pulse signal for every revolution of the crankshaft which is switched on to start the game and synchronises the avatar’s pedals with the real pedals. The VCO has an S-shaped characteristic, shown in (**c**), which limits the avatar’s speed to 0–12 m/s. The controls labelled *effort, offset* and *slope* are used to set the working range as indicated below the graph. The feint lines in (**d**) are the function where V is the voltage applied to the motor, T is the torque (N.m) and Ω is cadence (r.p.m.). The iCycle can deliver or absorb 30 W. **b** The iCycle is used with participants sitting in their own wheelchairs in front of the VR screen

The iCycle is described briefly in Fig. 1. The user remains seated in their wheelchair attached by adjustable straps to the iCycle, while their feet are strapped to footplates on the cranks (see Fig. 1b). The cycling cadence is set by a motor speed control, usually while the user remains relaxed, and sometimes until spastic responses have subsided. When the game is started, the user sees an avatar cyclist moving through a rolling landscape at a speed which depends on the torque they are producing. Our aim is to design a system that can be used independently at home. Usability was therefore critical and based on our laboratory studies we measured voluntary effort via torque rather than EMG amplitudes.

The main objective of this single-group pilot study was to investigate the effects of a short-term training programme using the iCycle on ISNC-SCI motor scores after incomplete SCI. Secondary objectives were to investigate the effects of iCycle training on cycling performance as well as outcome measures for voluntary function.

## Methods

**Fig. 2:**
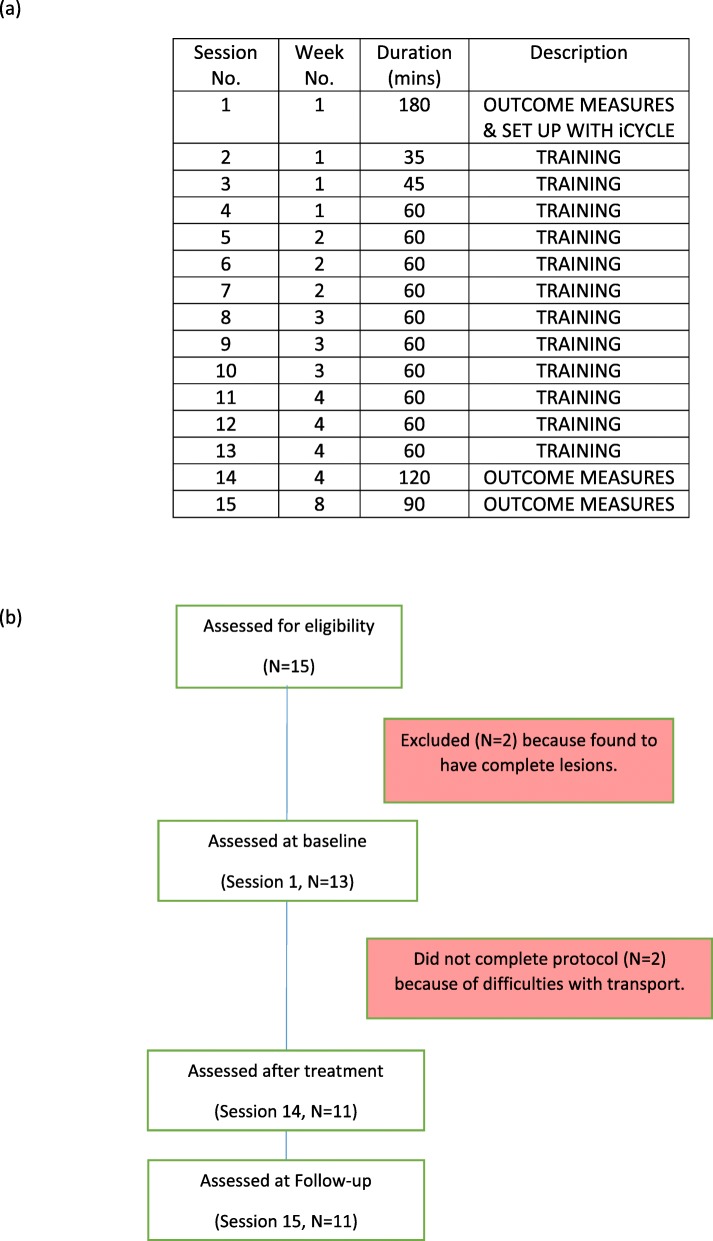
**a** Time schedule of outcome measures and intervention (**b**) CONSORT flow diagram

The aim of this study was to measure the effects of FES-cycle training combined with VR racing biofeedback on voluntary function. This study was carried out at the London Spinal Cord Injury Centre (LSCIC), Royal National Orthopaedic Hospital, Stanmore. Participants trained for three 1-h sessions per week over 4 weeks on the iCycle, which was set up in the therapy gym of the LSCIC. Outcome measures were assessed pre- and post-training, and 4 weeks after completing training (see Fig. 2a). Ethical approval for the study was provided by the City Road and Hampstead Research Ethics Committee (13/LO/0832), and all participants gave informed written consent prior to participating in the study (Additional files 1 and 2).

### Participants

Fifteen individuals with incomplete SCI (AIS B, C or D) aged 18–90 years and who were using a wheelchair for at least 2 hours per day were recruited. People were excluded if they had: a cardiac pacemaker; pressure sores or unresolved skin problems; unhealed lower limb fractures; pregnancy; active heterotrophic ossification (lower limbs); severe osteoporosis; complex regional pain syndrome; metal implants near electrode sites; lower limb malignancy; T6 and below spinal malignancy; uncontrolled autonomic dysreflexia; history of knee dislocation/subluxation; allergy to electrodes; cognitive difficulties; severe spasticity (Ashworth scale 4 or 5 in muscle groups that would prevent smooth pedalling) or neurological degenerative diseases. Participants were permitted to continue with their usual care throughout the study. Both inpatients and outpatients were recruited in order to optimise participant enrolment in the study.

### Outcome measures

Outcome measures were assessed by SCI-physiotherapists at baseline (B), end of training (EOT), and follow-up (FU), 4 weeks later. Voluntary motor function was assessed using ISNC-SCI motor scores. In this paper, these motor scores are bilateral (left and right added) which is also most common in the literature. Oxford scale motor power grading was carried out for knee extension/flexion and ankle plantarflexion/dorsiflexion, and the Modified Ashworth Score (MAS) was used to assess lower limb spasticity. Participants were asked to complete the Spinal Cord Independence Measure (SCIM), and walking ability was assessed using the Walking Index for Spinal Cord Injury (WISCI II) and 10 m-walk test.

### The iCycle

The iCycle (Fig. 1) is an FES ergometer, designed specifically for people with incomplete SCI. To encourage voluntary drive during pedalling, biofeedback is used through a VR game in which the speed of the avatar depends on the actual crankshaft torque while motion is maintained by a motor. The torque is measured on alternate revolutions of the pedals when there is no stimulation to confound the measurement. The difference between the average torque, while the participant is trying to propel the pedals, and its value while they are not, but with the legs being driven by the motor, is the voluntary drive. We assume that this voluntary drive is the same during revolutions with stimulation. After experiencing stimulation on alternate revolutions, this seems reasonable, but it is only an assumption. Because the avatar’s speed depends on the slope in the road, the voluntary drive must be increased if the virtual speed is to be maintained up hill. The unfolding scenery is interesting and further motivation is provided by competing (but virtual) cyclists, some of whom may be the participant in previous sessions.

### Setting up

During this trial, we set up the iCycle for each participant as follows. The height of the iCycle and the distance from the wheelchair were adjusted to be comfortable and safe, ensuring no pressure or friction points on the skin. The length of the cranks was adjusted to allow a smooth cycling motion; the wheelchair was then strapped to the iCycle. The virtual route was made a velodrome. The *effort gain* control and the *offset* (Fig. 1a) were adjusted so that with the participant relaxed, the speed was very slow, while, when they made their greatest effort, the lap time was 80–100 s. This gave all participants a similar virtual speed, regardless of their ability, at the start of training. For each participant, the set-up of the iCycle (including the *effort gain* and *offset*) were documented and not adjusted after training had started.

Electrical Stimulation (ES) sectors and amplitudes were set. ES was applied to the quadriceps, hamstring and gluteal muscles bilaterally. Electrodes (12.5 × 7.5 cm) were placed over the latero-proximal and medio-distal quadriceps and the proximal and distal midline of the hamstrings. Electrodes (7 cm diameter) were positioned proximally and distally on the midline of the gluteals. Biphasic stimulation was provided using two Odstock 4-channel stimulators (Odstock Medical Ltd., UK), with rectangular pulses 200 μs in width and at a frequency of 30 Hz. During the first session, minimum (first sensation of ES) and maximum stimulation amplitudes were determined for each participant, the latter defined as the highest level tolerated or maximum muscle contraction without overflow to antagonist muscles, whichever was lower. Stimulation on/off angles were set by manually moving the pedals to each of these 12 crankshaft angles (judged by the therapist) and setting that parameter. Stimulation angles and amplitudes were documented and could be altered during training.

### Training

Participants trained three times per week for 4 weeks (12 sessions in total). Total cycling time was gradually increased over the first three sessions from 20 to 30 then up to 45 min (depending on the participant’s exercise tolerance). Each training session consisted of; (i) a warm up (passive cycling followed by cycling with ES); (ii) velodrome laps: up to 5 virtual races around the velodrome (each separated by 2–3 min rest); (iii) free cycling: any virtual race(s) chosen by the participant and; (iv) a warm down. During the warm up, ES levels were gradually increased from each participant’s minimum to maximum levels, or to the maximum intensity tolerated on that day. VR feedback was provided to the participant during each velodrome lap and during free cycling. Participants were asked to provide Rates of Perceived Exertion (RPE) at the end of each velodrome lap, and at the end of their free cycling. During free cycling, if the participant repeated a previous session’s virtual race, their previous performance was displayed as a virtual competitor in the game, the aim being to provide inducement to go faster. Trainers also provided verbal encouragement throughout the velodrome laps and free cycling.

Throughout each training session, analogue signals from the iCycle (torque and shaft encoder signals) were sampled at 4 Hz and stored on a personal computer for offline analysis. The exact duration of cycling, the VR routes chosen, and other relevant clinical observations were recorded during each session using a training diary. Any adverse events were reported to Royal National Orthopaedic Hospital, and the chair of the Research Ethics Committee.

#### Data management and analysis

Each participant was given a unique ID, and data was pseudonymised at the point of collection. Participants were classified, according to time since injury at baseline, as sub-acute (< 12 months post-injury) or chronic (> 12 months post-injury). Individual data are presented, and median, range or interquartile range (IQR), unless stated otherwise. The correlation between change in ISNC-SCI motor scores (Follow-Up minus Baseline, FU-B) and other variables (age, time since injury, baseline ISNC-SCI motor score, baseline voluntary power output (PO), time spent training and stimulation amplitude) were analysed using Spearman’s Rank Order Correlation (IBM SPSS statistics 25).

Data collected from the iCycle were analysed using a custom script, written in Matlab. Torque data were averaged over each revolution and separated into stimulated and non-stimulated revolutions. Cadence was calculated using the shaft encoder signal over 15-s windows. Baseline torque and cadence was calculated from a 60-s window of passive cycling during the warm up period, where there was no evidence of spasms. Torque during the non-stimulated and stimulated revolutions were calculated by subtracting the baseline torque from the measured torque signal. PO was then calculated from the torque and cadence. Improvement in PO over time was analysed using Simple Linear Regression (IBM SPSS statistics 25).

The correlation between change in voluntary PO (slope from non-stimulated revolutions) and other variables (change in ISNC-SCI motor score, age, time since injury, baseline ISNC-SCI motor score, baseline voluntary PO, time spent training and stimulation amplitude) was analysed using Spearman’s Rank Order Correlation (IBM SPSS statistics 25).

## Results

Two of the 15 participants screened were not eligible and two withdrew from the study (see Fig. 2b). No data from the excluded participants was included in the analysis. Demographic details of the 11 participants who completed the study are provided in Table 1.

**Table 1:** Demographic data of the participants who completed the study

Participant	Group	Gender	Age	Injury Level	AIS Grade	Time since injury
1	C	F	67	T7	C	11y11m
2	C	M	80	T12	C	3y5m
4	C	M	58	T2	D	6y8m
5	C	M	55	C1	D	1y1m
6	C	M	73	C1	C	49y
7	C	M	58	T2	C	6y10m
9	SA	M	53	C3	C	0y2m
10	SA	M	67	C4	C	0y4m
12	SA	M	29	T2	C	0y5m
13	SA	M	21	T5	C	0y3m
15	SA	M	61	T3	D	0y2m

*C* Chronic, *SA* Sub-acute, *AIS* ASIA Impairment Scale

### Outcome measures

**Fig. 3:**
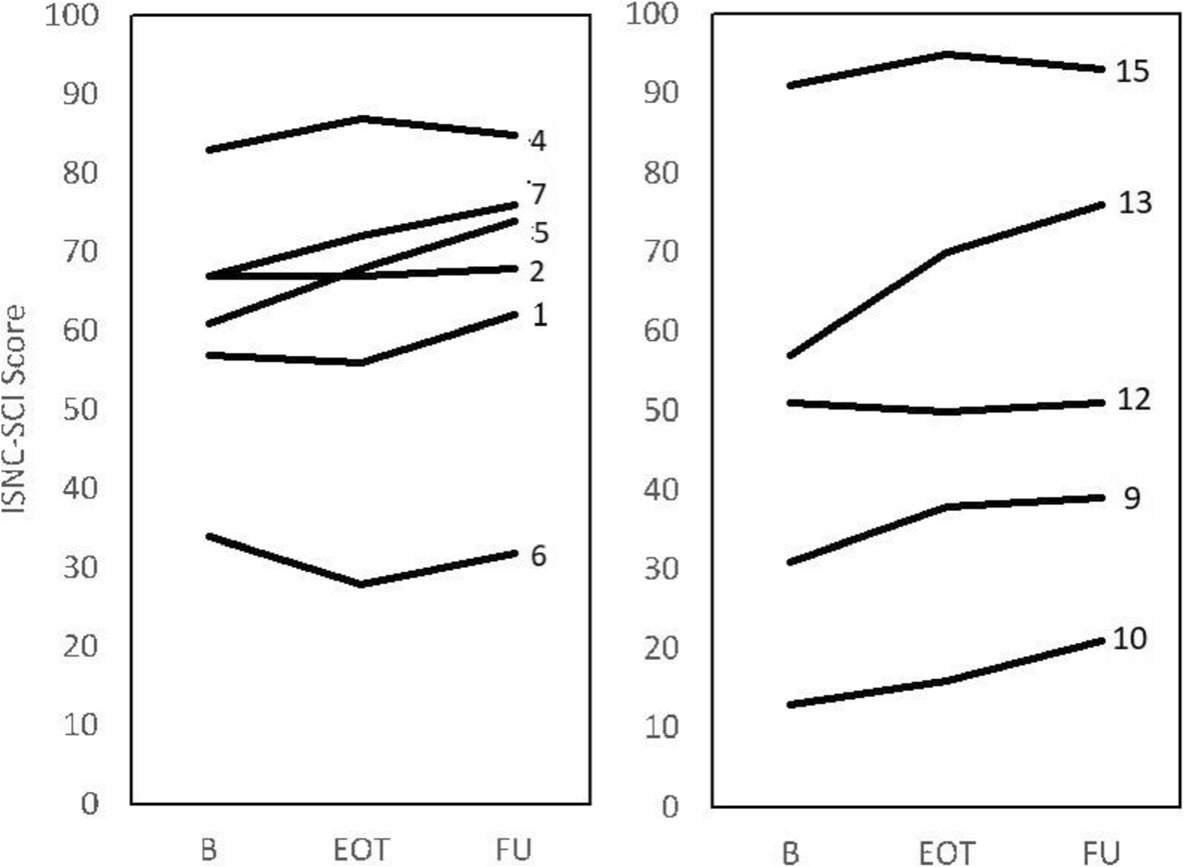
Change in ISNC-SCI motor scores at baseline (B), end of training (EOT) and follow up (FU). Participant number is provided beside each data set. Participants with chronic injuries are shown in the left panel and those with sub-acute injuries are shown on the right. The graph shows the changes on a scale from 0 (complete paralysis) to 100 (able-bodied)

**Fig. 4:**
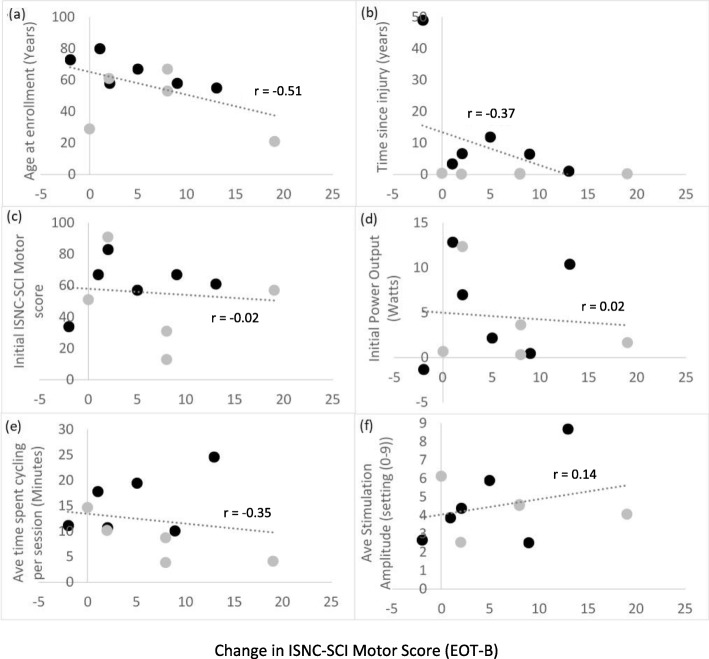
Change in ISNC-SCI motor scores at follow up (FU) relative to baseline (B), plotted against (**a**) age (years) at time of enrolment to the study, (**b**) time since injury (years), (**c**) initial ISNC-SCI score, (**d**) initial power output (watts), (**e**) cycling duration per session (min), (**f**) average stimulation amplitude (stimulator setting). Participants with chronic injuries are shown in black, those with sub-acute injuries in grey

ISNC-SCI motor scores for each participant at baseline, end-of-training and follow-up (FU) are shown in Fig. 3. The Median (IQR) improvement noted in the participants with chronic injuries (*n* = 6) was 3.5 (6.8) points; given their initial median ISNC-SCI motor scores were 64.0, the improvement was 10%. Of these six participants with chronic injuries, two improved by 8 points or more (Fig. 3). The Median (IQR) improvement among sub-acute participants (*n* = 5) was 8.0 (6.0) points. Initial median ISNC-SCI motor scores was 51.0, therefore the improvement was 16%. Of these participants, three improved by 8 points or more, and one (#13) improved by 19-points. Changes in ISNC-SCI motor score did not correlate with age (r = − 0.51; *p* > 0.05), time since injury (r = − 0.37, *p* > 0.05; Fig. 4b), baseline ISNC-SCI motor score (r = − 0.02, *p* > 0.05; Fig. 4c) or baseline PO during cycling (r = 0.02, *p* > 0.05; Fig. 4d). Change in ISNC-SCI motor score was also uncorrelated with training stimulus (time spent training, Fig. 4e, and stimulation amplitude, Fig. 4f; *p* > 0.05 for both).

Outcome measures for each participant are shown below: Oxford Scale (Table 2); MAS scores for the quadriceps, hamstrings and calf (Table 3); Total scores from the SCIM (Table 4), and a summary of these results (Table 5). Four of the five participants with sub-acute injuries, and one of the six participants with a chronic injury, had improvements in SCIM scores during training (Table 4). Only two of the participants included in the trial were ambulatory, therefore WISCI II scores and the 10 m walk test could only be completed for these participants (#7 and #15). Participant #7 showed no change in either measure, whereas Participant #15 demonstrated an improvement in WISCI II score of 5 points at EOT compared with Baseline, and 10 m walk test time improved from 82 s at baseline to 41 s at EOT.

**Table 2:** Oxford scale motor power charting for all participants at Baseline, End-of-Training (EOT) and Follow-Up (FU) for lower limb muscles

Participant		Flexion/Dorsiflexion						Extension/Plantarflexion					
		Right			Left			Right			Left		
		B	EOT	FU	B	EOT	FU	B	EOT	FU	B	EOT	FU
1	Knee	0	0	0	0	0	0	1	1	1	0	0	2
	Ankle	0	0	0	2	2	2	1	0	1	1	1	2
2	Knee	1	2	2	2	2	2	5	5	5	2	2	3
	Ankle	0	0	0	0	0	0	1	0	1	1	0	0
4	Knee	1	1	1	3	3	3	2	4	3	2	4	4
	Ankle	2	3	1	4	4	5	4	4	4	4	4	5
5	Knee	1	4	4	2	1	2	2	3	3	1	2	2
	Ankle	2	4	3	1	1	1	2	4	5	2	2	3
6	Knee	0	0	0	0	0	0	1	1	1	1	0	1
	Ankle	1	0	0	0	0	0	1	0	0	0	0	0
7	Knee	1	1	2	0	1	1	2	3	3	2	2	3
	Ankle	3	3	3	1	3	3	2	3	3	1	1	3
9	Knee	1	2	2	2	3	3	1	1	2	3	3	4
	Ankle	1	2	2	3	4	4	1	2	2	3	5	4
10	Knee	0	1	0	0	1	1	0	1	1	0	1	2
	Ankle	0	0	0	0	1	2	0	1	2	0	2	3
12	Knee	0	0	0	0	1	0	0	0	0	1	0	0
	Ankle	0	0	0	0	0	0	0	0	0	0	0	0
13	Knee	1	1	1	2	1	1	2	3	3	3	4	4
	Ankle	2	2	3	3	3	4	2	1	1	2	1	1
15	Knee	4	4	4	2	3	3	5	5	5	2	4	4
	Ankle	4	5	5	5	5	5	4	5	5	3	4	4

**Table 3:** Modified Ashworth Score (MAS) to assess lower limb spasticity at Baseline, End-of-Training (EOT) and Follow-Up (FU)

Participant	Quadriceps						Hamstrings						Calf					
	Right			Left			Right			Left			Right			Left		
	B	EOT	FU	B	EOT	FU	B	EOT	FU	B	EOT	FU	B	EOT	FU	B	EOT	FU
1	2	0	0	2	0	0	1	1	1	1	1	1	2	0	1	1	1	1
2	0	0	0	0	0	0	0	0	0	0	0	0	0	0	0	0	0	0
4	3	2	3	3	1	2	3	2	3	3	1	2	3	2	3	3	1	2
5	4	3	3	4	4	4	4	3	3	4	4	5	4	4	3	5	5	4
6	3	4	3	4	3	3	3	3	3	4	3	3	3	3	3	3	3	3
7	1	1	1	2	1	1	1	1	1	2	1	1	2	1	1	3	1	1
9	2	2	3	1	1	2	2	2	3	1	1	2	2	2	3	1	1	2
10	2	2	1	3	2	1	2	2	1	3	0	1	2	1	1	3	0	1
12	2	3	3	1	2	2	1	2	1	1	1	1	1	2	2	1	2	2
13	0	1	1	0	1	1	0	3	3	0	3	2	1	3	1	1	3	1
15	1	0	0	2	0	1	1	0	0	1	2	2	2	0	0	2	2	2

**Table 4:** Spinal Cord Independence Measure (SCIM) scores for all participants at Baseline, End-of-Training (EOT) and Follow-Up (FU) (data provided are summed across sub categories). Participants whose SCIM score changed during the study are shaded

Participant	SCIM		
	B	EOT	FU
1	67	67	67
2	68	68	68
4	74	74	74
5	43	44	45
6	54	54	54
7	69	69	69
9	19	20	20
10	10	11	16
12	71	71	71
13	74	75	77
15	70	83	83

**Table 5:** Summary of collected results for the outcome measures

Test	Measure of	Scale	Change from Baseline to Follow-up (all participants)		
				Median	Range
ISNC-SCI	SCI Classification	0–100		5.0	−2.0 -- + 19.0
Oxford	Strength	0–5	Knee Flex	0.5	−1.0 -- + 3.0
			Knee Ext	1.0	−1.0 -- + 2.0
			Ankle DF	0.5	−1.0 -- + 2.0
			Ankle PF	0.5	−1.0 -- + 3.0
SCIM	Independence: mobility	0–80		0.0	0.0 -- + 13.0
Ashworth (MAS)	Severity of spasticity	0–4	Quads	- 0.5	−2.0 -- + 1.0
			Hams	0.0	−1.0 -- + 3.0
			Calf	−0.5	−2.0 -- + 1.0

### Training

**Fig. 5:**
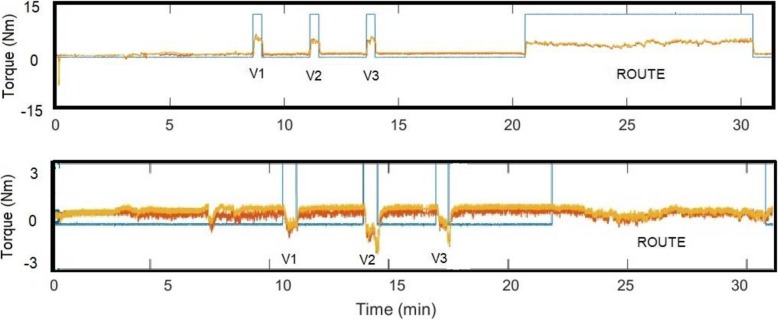
Raw plots from two participants during an iCycle session. The plots show torque data from the stimulated (yellow) and non-stimulated (orange) revolutions, and the game on signal (blue traces). The three velodrome laps (V1, V2, V3) and free cycling (route) are indicated. The upper panel represents a typical plot when no negative torque was recorded, and the lower panel represents a typical plot when negative torque occurred at the onset of voluntary effort

All 11 participants completed 12 training sessions, with no reported serious adverse events. An example raw torque trace taken from the iCycle after a single session is provided in Fig. 5 (upper panel), indicating baseline, three velodrome laps and one cross-country route completed by the participant. Average stimulation amplitude used by each participant on each muscle group across all training sessions is provided in Table 6. Average Rates of Perceived Exertion (RPE) for each participant across all sessions is also provided in Table 6.

**Table 6:** Average (SD) stimulation amplitude for quadriceps, hamstrings and gluteals, and Rates of Perceived Exertion (RPE) for each participant across all training sessions

Participant	Stimulation Amplitude			Rates of Perceived Exertion (RPE)	
	*Quadriceps*	*Hamstrings*	*Gluteals*	Laps	Free cycling
1	5.0 (1.4)	7.5 (0.6)	4.1 (0.2)	13.0 (0.4)	12.6 (1.4)
2	3.3 (0.6)	3.9 (1.1)	4.8 (1.0)	14.3 (0.9)	14.6 (1.8)
4	4.3 (0.6)	4.1 (0.3)	4.7 (0.5)	16.1 (1.1)	16.5 (0.8)
5	8.7 (0.6)	8.8 (0.4)	8.6 (0.6)	13.4 (0.8)	14.5 (1.2)
6	2.9 (0.4)	2.5 (0.3)	2.5 (0.3)	12.2 (0.8)	14.2 (0.6)
7	2.4 (0.4)	2.7 (0.4)	0.0 (0.0)	11.6 (0.6)	13.3 (0.5)
9	4.9 (0.5)	5.4 (0.9)	3.4 (0.6)	12.3 (0.4)	12.5 (0.5)
10	5.3 (1.0)	4.7 (0.8)	3.8 (0.7)	13.0 (0.5)	13.3 (0.8)
12	5.9 (1.6)	5.4 (1.4)	7.4 (1.0)	9.5 (0.6)	13.3 (0.8)
13	3.6 (1.2)	3.8 (1.2)	4.8 (1.7)	11.2 (0.8)	13.2 (0.9)
15	2.9 (0.3)	2.3 (0.5)	2.5 (0.4)	13.5 (0.9)	16.1 (1.4)

Stimulation amplitude is provided on an arbitrary scale (0–9), which represents 0-115 mA into a 1 k ohm load.

In 4 participants (#1, #6, #7 and #13), voluntary effort caused negative torque, as shown in Fig. 5 (lower panel), presumably due to spasticity. Negative torque was associated with voluntary intent to cycle rather than passive cycling, or FES cycling, since the torque was more negative during each velodrome lap and the cross-country route compared with passive cycling (baseline) and FES cycling (FES was provided throughout the session after baseline data had been collected).

### Velodrome laps

**Fig. 6:**
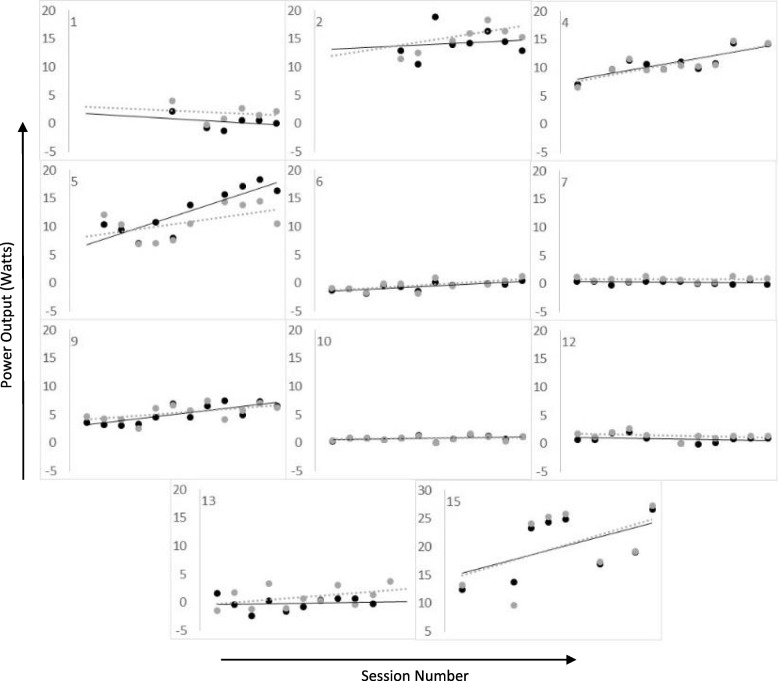
Power output from the stimulated (black) and non-stimulated (grey) revolutions during one velodrome lap for each participant across sessions 1–12

**Fig. 7:**
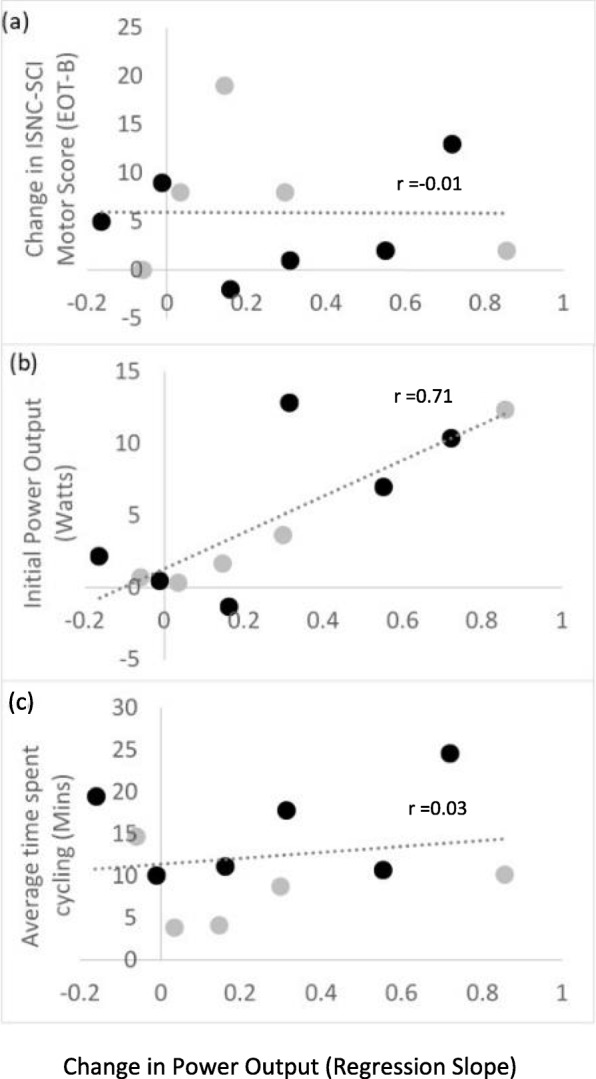
Change in power output (regression slopes taken from Fig. 6) plotted against (**a**) initial ISNC-SCI score, (**b**) initial power output (watts), (**c**) cycling duration per session (min). Participants with chronic injuries are shown in black, those with sub-acute injuries in grey

Each participant completed up to five virtual laps of the velodrome at the start of each training session (typically three were completed per session). Each of these laps was timed, and lap times were intended to provide session-to-session feedback to the participants. For each session, average PO during the fastest velodrome lap (from both the stimulated and non-stimulated revolutions) was calculated (Fig. 6). Some participants showed increases in PO with training whereas other did not. From the regression lines (Fig. 6), participants 2, 4 and 9 had a moderate improvement in PO with training (R^2^ > 0.3), and participants 5 and 15 showed a greater improvement in PO with training (R^2^ > 0.7). The remaining subjects showed little or no change. Improvement in PO did not correlate with change in ISNC-SCI motor score (r = 0.01, *p* > 0.05, Fig. 7a), but was related to their baseline cycling ability shown by a significant, positive correlation between slope and baseline PO (r = 0.71, *p* = 0.02, Fig. 7b).

### Free cycling

Average time spent free cycling during each session ranged from 4 to 25 mins. There was no correlation between time spent cycling and change in PO with training (r = 0.03, *p* > 0.05, Fig. 7c). Comments noted in the training diaries (by physiotherapists) included the occurrence of spasms during the session (*n* = 18), and participants’ anecdotal statements, as follows: enjoyment/pleased with the iCycle session (*n* = 5), improved standing when using a standing frame (n = 5), improved sleeping/sleeping well (*n* = 4), improved sensation (n = 4), increased alertness (n = 1), increased thigh size (n = 1) and improved ability to flex hip (n = 1). Two participants noticed skin redness at the end of a session, these were reported as adverse events.

## Discussion

This trial explored the effects of a 4-week, 12-session training programme using a novel FES bike, the iCycle, which used VR biofeedback to encourage voluntary effort. Improvements in ISNC-SCI motor scores were noted in both chronic and sub-acute participants (improvements ≥8 points in 5 out of the 11 participants); these improvements were unrelated to all other measured variables (Fig. 4). Moderate improvements in cycling PO were noted with training, but these were unrelated to improvements in ISNC-SCI motor scores: while some participants showed improvements in both PO and motor scores (e.g. #5), others showed considerable increases in ISNC-SCI motor score without any improvement in cycling PO (e.g. #7 and #13). Overall the results were variable, as shown by Table 5.

Among sub-acute participants, median improvement in ISNC-SCI motor score was 8.0 points (16% from B to FU), with 4 out of the 5 participants showing improvements. All of our sub-acute participants were between 2 and 5 months post-injury when enrolling in the study. The majority of natural recovery is known to occur during the first 6 months post injury [22–24]: Geissler et al [23] collected data from over 700 patients and showed changes of about 35 points for cervical and thoracic C & D patients in the 52 weeks after injury, nearly all in the first 26 weeks. It is therefore not possible to distinguish the effect of natural recovery from neurological recovery due to iCycle training in the first year. However, the improvements we noted occurred over a relatively short period (8 weeks), therefore a large RCT is warranted to verify whether iCycle training increases natural recovery rate in sub-acute participants. Functional improvement was noted in SCIM scores, from 8 at B to 13 at EOT and retained at FU (Table 4), and walking in the only ambulatory sub-acute participant [15] improved from walking with a frame and one person to assist, to a frame without assistance.

Among the participants with chronic injuries, median improvement in ISNC-SCI motor score was 3.5 points (10% from B to FU). Yaşar et al. [14] tested FES cycling without voluntary effort (participants were told *not* to contribute voluntarily to the cycling) in ten people with chronic AIS C & D injuries and found similar median improvement in motor score (3.0 points) but after 3 months training as opposed to 1 month in our study. Therefore, using feedback on voluntary effort in our study may have increased the rate at which changes occurred. Our findings can also be compared to motor recovery seen after gait training for AIS C & D people. Morrison et al. [25] reported ISNC-SCI scores from 60 participants (0.1–45 years post injury) doing 120 sessions of body-weight supported locomotor training. The training time averaged (SD) 11.3 (9.3) months (sessions every 3 days) and the mean improvement in lower-limb motor scores was 6, suggesting that recovery rate (as assessed by ISNC-SCI) is slower with gait training than iCycle training. Recently, Wagner et al. [26] reported greater motor recovery on 3 participants with chronic incomplete SCI (AIS C & D) who carried out overground walking training combined with epidural stimulation for 5 months (the number of training sessions was not stated). At the end, their motor scores increased by 16, 11 and 4 respectively (mean 10.3).

Among chronic participants, motor recovery can be assumed to be related to the intervention, although the placebo effect of taking part in a research study should not be discounted. Improvements in motor function of 8 points or higher (as noted in Participants #5 and #7) indicated clinically important gains in neurological and muscle function [27–29]. Other functional improvements were also noted in these participants: 2-point increase in SCIM score (#5) and anecdotal report of improved standing when using a frame (#7).

Improvements in ISNC-SCI motor score appeared to be unrelated to age, but the two chronic participants who experienced little or no improvement in motor scores (participants #2 and #6) were the oldest (both > 70 years); in these participants recovery may have been limited by atrophy of the musculoskeletal system known to occur with aging [30]. Improvements in ISNC-SCI motor score also appeared to be unrelated to injury classification or time since injury (Fig. 4b-d). However, there may be injury-related factors that are not captured by the ISNC-SCI scoring system that explain why some participants appeared to respond to the intervention where others did not, such as the presence of non-functional residual pathways crossing the lesion site.

Our original idea was that neuroplasticity would be enhanced by temporal correlation of voluntary effort and electrical stimulation of the paralysed or paretic muscles, based on Hebbian learning. This hypothesis is not supported by the poor correlation between stimulation intensity and improvement in ISNC-SCI (Fig. 4f). FES is different from the more general “activity-based restoration” advocated by Sadowsky & McDonald [31]. It is of great practical importance to know what the relative contributions to recovery are from muscle stimulation, from motor-driven leg motion, and from voluntary effort.

### Cycling performance

In some subjects moderate increases in peak cycling PO were noted at EOT compared to B (up to a maximum of 14 W, see Fig. 6). These are similar to the relatively small improvements that have been reported previously after FES cycle training [6], which are attributable to observed improvements in lower limb muscle size [13, 32–34] and strength [6, 13, 32]. We observed a moderate correlation (R^2^ = 0.64, Fig. 7b) between baseline PO (from the non-stimulated revolutions) and the change in PO with training, indicating that the subjects with higher voluntary power at baseline were more likely to improve with training.

The average PO measured over a single velodrome lap was somewhat variable across sessions, ranging from − 1.9 W to + 27.3 W during stimulated revolutions and − 2.3 W to + 26.6 W during non-stimulated revolutions. Previous FES cycling studies report POs of similar magnitude (0-35 W [35]), however, the majority of previous work has been in people with complete injuries, which usually allows for high stimulation currents (~ 60-100 mA is typically used). Our participants had variable sensory impairments, which restricted stimulation current levels. The additional FES had little impact on PO, perhaps due to the rapid fatigue caused by FES [35].

Negative POs during training (FES combined with voluntary effort, see Fig. 5, lower panel) were an unexpected finding: negative torque was not due to passive resistance (e.g. weight of the limbs) as this had been corrected for. The negative PO was principally due to co-contraction of lower limb muscles at the start of the VR game (FES had already been switched on at this point), perhaps due to spasms. This was additionally documented by physiotherapists in the training diaries. Presumably, FES combined with voluntary effort facilitated central nervous system excitability [36], which may have provoked antagonistic co-contraction, due to impaired reciprocal inhibition in people with incomplete SCI [37, 38]. For example, during active pedalling, normal modulation of the soleus H-reflex has been reported to be reduced or absent in people with incomplete SCI due to loss of supraspinal control over inhibitory spinal mechanisms [37].

The commercially available software used for training offered many different VR environments, including mountainous or flat terrain. During training, participants were able to view their previous performance by means of a virtual competitor in the software programme, and reported that this was particularly motivating. Time spent cycling in different VR environments varied across participants, ranging from approximately 5–25 min. There was no correlation between training duration and improvements in ISNC-SCI motor scores, or PO during cycling.

### Limitations & future work

This was a small pilot study of a novel device, which did not include a control group. The study has identified where technical improvements are needed, and informed choice of outcome measures. The use of PO alone as a measure of recovery is unsatisfactory because it may be confounded by muscle-training effects. In future, functional outcome measures should be combined with measures that provide insight into underlying neuroplastic mechanisms such as motor-evoked potentials to assess changes in the motor pathway. Results were variable and larger, better-controlled clinical trials are needed to prove that the addition of VR biofeedback increases the rate of recovery from FES cycling. The dose of cycling was not equal for all participants, either in minutes or pedal revolutions; this could be changed in future if this restriction on the participants was considered important. From a clinical perspective, the aim of rehabilitation is to challenge patients to extend their ability. As each patient has a different ability level, to be optimised clinically, both time and pedal revolutions need to be variable both within and between patients. The long-term objective of the work is to develop a device and protocol that are clinically useful, rather than to perform a purely scientific experiment.

There were some limitations of the iCycle, which we propose to address before conducting further clinical trials. Some arrangement is required so that participants who produce negative torques are not disincentivised. We found that weak participants produced torques that were too small compared to the background interference: this may be improved by better filtering of the torque signal, or perhaps by using EMG during the non-stimulated revolutions. The biofeedback system may also be improved by optimising the VR environment, for example by introducing remote virtual races and/or creating a more immersive VR environment. The participants in this study were particularly encouraged by racing against their previous performance, displayed as a virtual competitor, therefore introducing a multi-player environment where participants are able to compete against other SCI participants within a virtual race might also provide an effective stimulus to encourage voluntary effort and participation.

## Conclusions

The iCycle is an innovative progression of traditional FES cycling systems: it provides VR biofeedback based only on the voluntarily generated torque to encourage voluntary effort. Following a 4-week intervention using the iCycle, improvements in ISNC-SCI motor scores were noted in both chronic and sub-acute participants (improvements ≥8 points in 5 out of the 11 participants). Our data suggest that recovery rate may be faster when voluntary effort is combined with FES cycling with VR biofeedback. Larger controlled trials are needed to verify these findings and to understand the mechanisms of effect.

## Supplementary information

**Additional file 1.** Participant Information Sheet.

**Additional file 2.** Consent Form.

## Data Availability

The datasets used and/or analysed during the current study are available from the corresponding author on reasonable request.

## Abbreviations

AIS: ASIA Impairment Scale
B: Baseline
EOT: End of Training
FES: Functional Electrical Stimulation
FU: Follow up
ISNC-SCI: International Standards for Neurological Classification of Spinal Cord Injury
LSCIC: London Spinal Cord Injury Centre
MAS: Modified Ashworth Scale
PO: Power Output
SCI: Spinal Cord Injury
SCIM: Spinal Cord Independence Measure
SD: Standard Deviation
TMS: Transcranial Magnetic Stimulation
VR: Virtual Reality
WISCI II: Walking Index for Spinal Cord Injury II
