# Supplementary material for: The effects of FES cycling combined with virtual reality racing biofeedback on voluntary function after incomplete SCI: a pilot study
Source: J Neuroeng Rehabil. 2019 Nov 27;16:149. doi: 10.1186/s12984-019-0619-4 (PMC6880599; doi:10.1186/s12984-019-0619-4)
Supplement: Supplementary file 1 — Additional file 1. Participant Information Sheet. [file 12984_2019_619_MOESM1_ESM.pdf]

Royal National Orthopaedic Hospital
NHS Trust

RNOH Stanmore
Brockley Hill
Stanmore
Middlesex
HA7 4LP

Participant information sheet for in- and outpatients

Development and testing of an FES bike for an experiment in functional recovery after spinal cord injury

We would like to invite you to take part in a research study being undertaken by Royal National Orthopaedic Hospital Physiotherapy Department. Before agreeing to take part, it is important for you to understand why the research is being carried out and what it will involve. Please take your time to read the following information sheet carefully and discuss it with friends and relatives if you wish. If you have any questions or anything is not clear, please contact us at the email address or telephone number given at the end of this information sheet.

What is the purpose of this study?

Individuals with incomplete spinal cord injuries (SCI) have the potential for some degree of nerve recovery. Research has demonstrated that recovery can be enhanced by the use of Functional Electrical Stimulation (FES) to the muscles. This study will look at whether more recovery can be made by using a combination of FES and voluntary effort to turn the pedals of a static bike (FES bike). To encourage people to use as much voluntary effort as they can, they will participate in a virtual reality cycle race displayed on a laptop. The harder they pedal the better they do in the race. This initial phase of the study is to assess the feasibility of using the bike including getting feedback from participants and therapists.

Why have I been chosen?

We would like sixteen to twenty people with incomplete SCI to participate in this study (six to ten inpatients and ten outpatients). You have been identified as a possible participant.

Do I have to take part?

You do not have to take part in the study. If you decide to take part you are free to withdraw at any time and without giving a reason. A decision to withdraw at any time, or a decision not to take part, will not affect your rights or the care you receive.

What will happen to me if I take part?

A member of the physiotherapy administration team will contact you in person one week after receiving this information sheet. They will ask whether you would like to take part in the study. If you express an interest, the lead researcher (who is a qualified physiotherapist) will arrange to contact you to discuss your participation in the study and answer any questions you have.

If, following this, you would like to participate your first appointment will be arranged with the lead researcher in the physiotherapy gym of the London Spinal Cord Injuries Centre at Stanmore. The researcher will ask you to give written informed consent to taking part. They will also ask for your consent to be photographed and for videos to be taken while you are using the bike.

The researcher will then perform some simple tests and ask you some questions to ensure you satisfy the criteria for taking part in the study. One of the tests will be to check that your muscles respond to electrical stimulation without giving you undue discomfort.

There are 2 parts to this study. You may be invited to take part in Part I or Part II, or both parts.

PART I

The first part of the study will assess the feasibility of using the FES bike. If you are participating in this part of the study, you will be required to attend two sessions using the FES bike on two consecutive weeks.

Session 1 (1 ½ - 2 hours)	Give written informed consent.<br>First session using FES bike.
Session 2 (1 ½ - 2 hours)	Second session using FES bike.<br>Complete semi-structured interview to provide feedback about the FES bike.

PART II

Six to ten participants will be invited to participate in the second part of the study. This part will allow us to assess whether the treatment is effective. If you are participating in part two of the study, you will be required to attend fifteen sessions; twelve of them will be training sessions (up to three sessions per week over a period of four consecutive weeks). You will also complete three outcome measures/assessment sessions (before you start your FES bike sessions, after your final FES bike session and then after four weeks from your last training session.

Session 1: (3 hours - can be spread over two visits if you prefer)	Complete outcome measures/assessments (ASIA (American Spinal Injury Association) neurological classification, lower limb strength, SCIM (Spinal Cord Independence Measure), spasticity assessment, physiology assessment (TMS)).<br>Show you the iCycle and get you set up for training.
Sessions 2-13: (1 hour each)	FES Bike sessions (12 in total)
Session 14: (2 hours)	Complete outcome measures/assessments (same as above).<br>Complete semi-structured interview to provide feedback about the FES bike.
Session 15: (1.5 hours)	Complete outcome measures/assessments (same as above).

There will be scheduled breaks during each session but you may take as many breaks as you wish during the sessions.

Out of pocket expenses will be reimbursed for outpatients.

With your consent, your GP will be informed that you are participating in this study.

What will I have to do?
We will show you the FES bike and the virtual reality cycle race game; explain how it works and what we want you to do. You will use the FES bike from a seated position in your own wheelchair. The bike will be adjusted to suit you. Large self-adhesive electrodes, which provide the electrical stimulation, will then be attached to the skin over the muscles of both legs (see appendix 1 for potential electrode placement). You will be asked always to wear loose fitting, comfortable clothing.
Before you use the FES bike we will test your response to stimulation and set the limits to ensure you do not experience undue discomfort. Your legs will then be strapped onto the bike and stimulation levels will be adjusted to generate appropriate movement. After a few 'test runs' you will be able to participate in a virtual reality cycle race seen on a laptop screen. You will be asked to try as hard as possible to turn the cycle pedals. The harder you push, the faster the virtual bike will go. The length of the race will depend on the strength and endurance of your muscles and your cardiovascular fitness, but is expected to last 20-45 minutes.

We will also carry out a semi-structured interview with you to find out your opinions on the FES bike.

For participants in Part two only:
If you are participating in part two of the study, you will be asked to rate how heavy and strenuous the exercise feels to you and how tired you are. You will also carry out the outcome measures/assessments described in the Table above. The Physiology assessment (TMS) is optional. If you do take part, we will measure your nerve connections using a device that sends a small pulse from your brain to the muscles in your leg. By measuring the response in your muscles we can tell how good the connections are.
What kind of personal information is needed and how is it going to be used?
The researchers may inspect your medical records to obtain data about your injury for example, but all information about you will be kept confidential by using a unique identifier (ID) code.

What are the possible benefits of taking part?
We do not know whether you will benefit from taking part in this study. However, we do expect that the results will inform researchers working in the field of spinal cord injury. The aim is that this study will lead to further studies into the use of this equipment.

What happens when the research study stops?
Outpatients who have not previously used an FES bike will be offered the opportunity to be placed on the LSCIC physiotherapy outpatient waiting list for a 6 week programme using an RT300 FES bike (this does not have a virtual reality cycle game linked to it).

Inpatients will have the opportunity to continue using the RT300 FES bike during their hospital admission as part of their rehabilitation if their named physiotherapist considers it to be appropriate. However, the frequency of these sessions may be less than during the research study.

What are the side effects of taking part?
1. FES and cycling can be tiring. This could potentially impact on other aspects of your day-to-day function throughout the time you are participating in the study.
2. FES may cause a little discomfort, especially for those with sensory incomplete injuries. Upper limits of stimulation will be set to avoid undue discomfort.
3. There is a small risk of reddening or irritation of the skin under the electrodes. Slight reddening which fades after about 10 minutes is normal. Participants will be asked to inform the therapists immediately if they notice prolonged skin reddening or soreness. In rare cases participants may not be able to continue in the study or may need to take a break of a few days before re-commencing once their skin reaction has resolved.

4. There is a small risk that FES could trigger autonomic dysreflexia. Autonomic dysreflexia can cause a sudden increase in blood pressure and a severe headache, which if left untreated can result in stroke or death. If you experience the symptoms of autonomic dysreflexia the FES will be discontinued. If you are an outpatient, you will be advised to take your medication for autonomic dysreflexia if symptoms do not resolve. Therefore, if you have a T6 injury and above you should bring appropriate medication to manage autonomic dysreflexia to all your appointments. If you are inpatient, you will be returned to the ward immediately, where medication will be given if required.

What if something goes wrong?
If you have a concern or a complaint about this study you should contact Research & Development Department (research@rnoh.nhs.uk) at RNOH, Brockley Hill, Stanmore, Middlesex, HA7 4LP; Tel: 0208 909 5529 . If you remain unhappy and wish to complain formally Research & Development Department will provide you with details of the relevant Complaints Procedure.

In the event that something does go wrong and you are harmed during the research and this is due to someone's negligence then you may have grounds for legal action for compensation against the study sponsor, but you may have to pay your legal costs. The normal NHS complaints mechanisms will still be available to you and you will be advised how to proceed with any claims.

If you feel you would like psychological support in relation to this study, the London Spinal Cord Injuries Centre psycho-social team can be contacted on 0208 909 5521 (Dr Carol Smyth).

What if new information about risks or side effects becomes available during the study?
Sometimes during the course of a research project, new information becomes available about the treatment that is being studied. If this happens, the lead researcher will inform you about it and discuss with you whether you want to continue taking part in the study.

Who is organising and funding the research?
The study is being organised by the Royal National Orthopaedic Hospital, Stanmore, in collaboration with University College London and The University of Southampton. Funding for this study is through a grant from the charity INSPIRE (www.inspire-foundation.org.uk).

Who has reviewed the study?
The scientific panel of the grant awarding body 'INSPIRE' reviewed the study. In addition, all research in the NHS is reviewed by an independent group of people called a Research Ethics Committee, who have a responsibility to protect your interests. This study has been reviewed and given favourable opinion by the NRES Committee London – City Road and Hampstead.

What will happen to the results of the research?
The results will be presented at meetings and may be presented at conferences or published in research papers for scientific journals. The results will also provide a stepping stone towards further studies using this equipment. We will send you a lay summary of our findings at the end of the study if you request this.

Will my taking part in this study be kept confidential?
Yes. All information that is collected about you during the course of the research will be kept strictly confidential. Any information about you that is used in research reports, publications or presentations will be anonymised and refer only to your study ID. Photographs and videos may be taken if you have given consent.

Contact for further information:
If you would like more information please contact Sue Paddison or Sandra Bulpitt on 0208 909 5500 or email sandra.bulpitt@rnoh.nhs.uk.

Thank you again for taking the time to read this information.

Appendix 1: Diagram showing potential FES electrode placement

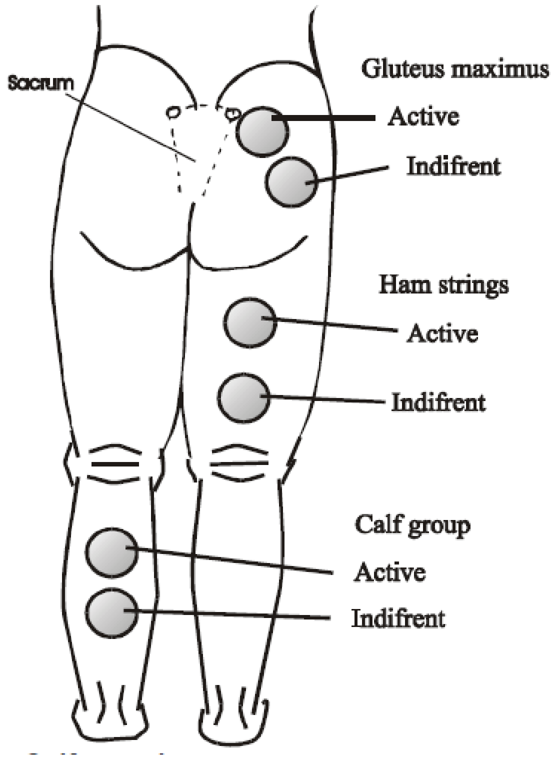

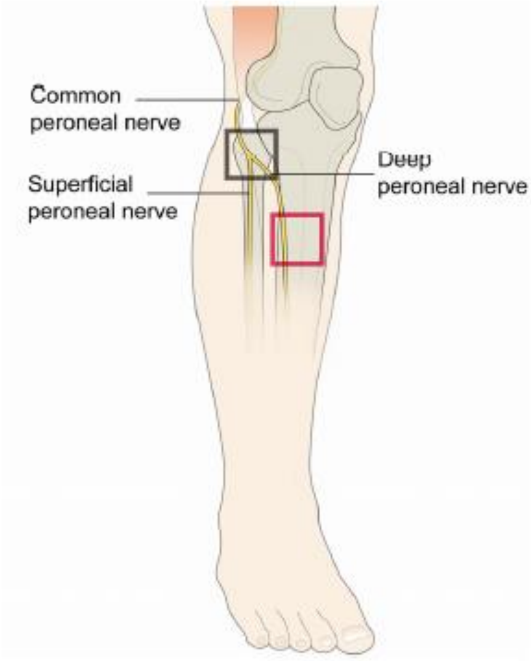
Common
peroneal nerve
Superficial
peroneal nerve
Deep
peroneal nerve
