# Supplementary material for: The effects of FES cycling combined with virtual reality racing biofeedback on voluntary function after incomplete SCI: a pilot study
Source: J Neuroeng Rehabil. 2019 Nov 27;16:149. doi: 10.1186/s12984-019-0619-4 (PMC6880599; doi:10.1186/s12984-019-0619-4)
Supplement: Supplementary file 2 — Additional file 2. Consent Form. [file 12984_2019_619_MOESM2_ESM.pdf]

Royal National Orthopaedic Hospital
NHS Trust

RNOH Stanmore
Brockley Hill
Stanmore
Middlesex
HA7 4LP

Patient Identification Number for this trial:

CONSENT FORM

Title of Project: Development and testing of an FES bike for an experiment in functional recovery after spinal cord injury

Name of Lead Researcher: Sue Paddison

Please initial box

1. I confirm that I have read and understand the participant information sheet dated _________________ (version) for the above study. I have had the opportunity to consider the information, ask questions and have had these answered satisfactorily. ☐

2. I understand that my participation is voluntary and that I am free to withdraw at any time without giving any reason, and without my medical care or legal rights being affected. ☐

3. I understand that relevant sections of my medical notes and data collected during the study, may be looked at by individuals responsible for the research, from regulatory authorities or from the NHS Trust, where it is relevant to my taking part in this research. I give permission for these individuals to have access to my records. ☐

4. I agree to take part in the Physiology (TMS-specific) measurements ☐

5. I agree to my GP being informed of my participation in this study. ☐

6. I would like to receive a copy of the summary of results from this study. ☐

7. I agree to take part in the above study. ☐

_________________________	________________	_________________________
Name of Patient	Date	Signature
_________________________	__________________	_________________________
Name of Person taking consent	Date	Signature

Forms: 1 for patient; 1 (original) for research site file; 1 to be kept in medical notes.
